# Melatonin-Producing Endophytic Bacteria from Grapevine Roots Promote the Abiotic Stress-Induced Production of Endogenous Melatonin in Their Hosts

**DOI:** 10.3389/fpls.2016.01387

**Published:** 2016-09-21

**Authors:** Jian Jiao, Yaner Ma, Sha Chen, Chonghuai Liu, Yuyang Song, Yi Qin, Chunlong Yuan, Yanlin Liu

**Affiliations:** ^1^College of Enology, Northwest A&F UniversityYangling, China; ^2^Zhengzhou Fruit Research Institute, Chinese Academy of Agricultural SciencesZhengzhou, China; ^3^Institute of Chinese Materia Medica, China Academy of Chinese Medical SciencesBeijing, China

**Keywords:** melatonin, endophytic bacteria, UPLC-MS/MS, grapevine, abiotic stress

## Abstract

Endophytes form symbiotic relationships with plants and constitute an important source of phytohormones and bioactive secondary metabolites for their hosts. To date, most studies of endophytes have focused on the influence of these microorganisms on plant growth and physiology and their role in plant defenses against biotic and abiotic stressors; however, to the best of our knowledge, the ability of endophytes to produce melatonin has not been reported. In the present study, we isolated and identified root-dwelling bacteria from three grapevine varieties and found that, when cultured under laboratory conditions, some of the bacteria strains secreted melatonin and tryptophan-ethyl ester. The endophytic bacterium *Bacillus amyloliquefaciens* SB-9 exhibited the highest level of *in vitro* melatonin secretion and also produced three intermediates of the melatonin biosynthesis pathway: 5-hydroxytryptophan, serotonin, and *N*-acetylserotonin. After *B. amyloliquefaciens* SB-9 colonization, the plantlets exhibited increased plant growth. Additionally, we found that, in grapevine plantlets exposed to salt or drought stress, colonization by *B. amyloliquefaciens* SB-9 increased the upregulation of melatonin synthesis, as well as that of its intermediates, but reduced the upregulation of grapevine tryptophan decarboxylase genes (*VvTDC*s) and a serotonin *N*-acetyltransferase gene (*VvSNAT*) transcription, when compared to the un-inoculated control. Colonization by *B. amyloliquefaciens* SB-9 was also able to counteract the adverse effects of salt- and drought-induced stress by reducing the production of malondialdehyde and reactive oxygen species (H_2_O_2_ and O_2_^-^) in roots. Therefore, our findings demonstrate the occurrence of melatonin biosynthesis in endophytic bacteria and provide evidence for a novel form of communication between beneficial endophytes and host plants *via* melatonin.

## Introduction

Melatonin (*N*-acetyl-5-methoxytryptamine) was first isolated from the bovine pineal gland (Lerner et al., 1958) and is now recognized as ubiquitous among living organisms, including humans, animals, plants, bacteria, fungi, and macroalgae (Tilden et al., 1997; Rodriguez-Naranjo et al., 2012; Tan et al., 2012). In vertebrates, the molecule functions as a biological modulator of mood, sleep, sexual behavior, seasonal reproductive physiology, and circadian rhythms (Reiter, 1993; Hardeland, 2008; Reiter et al., 2010); as a potent antioxidant, with free radical-scavenging activities; and as a stimulator of antioxidant enzyme activities (Reiter et al., 2005). Meanwhile, the occurrence of melatonin in higher plants wasn’t documented until almost 50 years later, when it was reported by both Dubbels et al. (1995) and Hattori et al. (1995). Since then, melatonin has been recognized to function as an abiotic antistressor, by protecting plants against ROS that are produced as a result of harmful abiotic stresses (Posmyk et al., 2008, 2009; Arnao and Hernández-Ruiz, 2009a; Nawaz et al., 2016). In addition, melatonin also functions as a plant regulator, with growth-promoting effects similar to those of IAA, which is a plant hormone in the auxin class (Hernández-Ruiz and Arnao, 2008).

Since the discovery of melatonin in higher plants, the factors that influence the endogenous melatonin levels in plants have remained relatively unexplored. Recently, reports have demonstrated that different abiotic stressors are able to elevate the endogenous melatonin levels of plants (Arnao and Hernández-Ruiz, 2013a,b; Shi et al., 2015; Hernández-Ruiz and Arnao, 2016) and that stress-induced ROS bursts may be the common factor that triggers the accumulation of melatonin (Arnao and Hernández-Ruiz, 2015). Under natural conditions, the internal organs of plants are frequently colonized by a vast number of diverse microbes that are able to interact with their hosts and, thereby, modulate plant growth and development (Sturz et al., 2000). Soil microbes, for example, have been shown to enter and proliferate within plant roots (Hardoim et al., 2008), and in grapevines, naturally occurring endophytes have been isolated from roots, stems, leaves, and various reproductive tissues (e.g., inflorescences, seeds, and fruits; Compant et al., 2011). These symbiotic organisms are important in defending their hosts against phytopathogens (Lindow and Brandl, 2003; West et al., 2010) and may also promote the growth of their host plants *via* nitrogen fixation (Elbeltagy et al., 2001), phosphorus solubilization (Richardson et al., 2009), and the enhancement of plant hormones levels (Ali et al., 2009). Therefore, endophytes are generally recognized as important and beneficial components of plant micro-ecosystems. In fact, since endophytes often supply their hosts with plant hormones, we speculate that endophytes are capable of producing melatonin and that they provide melatonin to their plant hosts. This conjecture is based on observations that (i) melatonin has been identified in microorganisms, such as aerobic photosynthetic bacteria (Tilden et al., 1997), recombinant *E. coli* (Byeon and Back, 2016) and some fungi (Manchester et al., 1995; Hardeland and Poeggeler, 2003); (ii) melatonin biosynthesis is likely to be evolutionarily conserved (Tan et al., 2014); (iii) the cellular machinery for melatonin synthesis in eukaryotes may have been inherited from bacteria, as a result of endosymbiosis (Tan et al., 2013); and (iv) bioinformatic analyses has revealed that enzymes involving in melatonin synthesis occur in bacterial genomes (Pavlicek et al., 2010; Falcón et al., 2014). However, to the best of our knowledge, the ability of endophytic bacteria to produce melatonin has not been reported, and the synthetic pathway of melatonin in heterotrophic bacteria remains to be elucidated.

Therefore, in the present study, we used a culture-dependent method to isolate endophytic bacteria from the roots of three grapevine varieties and screened the resulting cultures for their *in vitro* capacity to produce melatonin. We subsequently used a promising endophytic strain that produced high levels of melatonin to investigate the intermediates of the melatonin biosynthesis pathway, and in addition, we also performed root colonization experiments, in order to evaluate the effect of the strain on the endogenous melatonin production of host plants under abiotic stress. Finally, to examine whether stress-induced changes in melatonin levels were associated with the induction of melatonin synthesis, we performed qRT-PCR analysis of *VvSNAT* and several *VvTDC*s genes, both of which play a pivotal role in regulating melatonin biosynthesis in plants (Byeon et al., 2013; Zhao et al., 2013).

## Materials and Methods

### Isolation of Endophytic Bacteria from Grapevine Roots

We selected three grapevine varieties for our experiments, including the Chinese wild grapevine *Vitis amurensis* ‘Changbai 9,’ *V. vinifera* ‘Cabernet Sauvignon,’ and *V. labruscana* ‘Summer Black,’ and collected root samples from plants that were cultivated at the National Grape Germplasm Repository (113°70′ E; 34°72′ N), Zhengzhou Fruit Research Institute, Chinese Academy of Agricultural Sciences, Zhengzhou, Henan, China. The root samples were collected from one vine of each variety, when the vines were in flower, and a total of 5 g roots were collected from five randomly chosen root sections (∼20 cm below ground) each plant and pooled in sterile 15-mL tubes. The pooled root samples were then kept in refrigerated boxes and transported to the laboratory within 1 h of collection for subsequent processing.

The roots were surface-sterilized in 70% ethanol for 3 min, followed by soaking in sodium hypochlorite (3% available chlorine) for 2 min, and were then rinsed three times with sterile water. Next, each sample was ground and homogenized in 1.5-mL of PBS using sterile quartz sand in individual mortars. The resulting homogenate was serially diluted (10^-3^, 10^-4^, and 10^-5^) and plated on nutrient agar (3 g/L beef extract, 10 g/L tryptone, 5 g/L NaCl, and 20 g/L agar, pH 7.4) with 100 mg/L actidione to inhibit the growth of fungi, and each dilution was prepared in triplicate. In parallel, we also checked the efficiency of our surface sterilization procedure by plating 100 μL of the last washing solution (i.e., sterile water used for third rinse) onto nutrient agar. Then, after incubation at 28°C for 2–3 days, the number of colony-forming units was counted, and four to six representative isolates of each morphology were collected and purified by streaking onto fresh nutrient agar plates. Each purified isolate was maintained at -80°C in 1 mL sterile nutrient broth that contained 20% glycerol.

### Genomic DNA Extraction and Species Assessment

DNA extraction was performed using the TIANamp bacteria DNA Kit (Tiangen, Beijing, China), according to the manufacturer’s instructions, and in order to identify each of the isolates, we amplified the 16S rRNA sequence, using the primers 8F (5′-AGAGTTTGATCCTGGCTCAG-3′) and 1063R (5′-ACGGGCGGTGTGTRC-3′) (Wang and Qian, 2009), as well as the *gyrB* sequence, which encodes the B subunit of the type II topoisomerase DNA gyrase, using the degenerate primers UP-1 (5′-GAAGTCATCATGACCGTTCTGCAYGCNGGNGGNAARTTYGA-3′) and UP-2r (5′-AGCAGGATACGGATGTGCGAGCCRTCNACRTCNGCRTCNGTCAT-3′) reported by Yamamoto and Harayama (1995). The resulting PCR products were purified and bi-directionally sequenced, using the same primers that were used for PCR amplification. The sequences were then compared with reference sequences in GenBank, using the online Blastn software^1^, and identification was considered valid when the identity of a contiguous sequence was ≥99%.

### Screening for Melatonin-Producing Endophytic Bacteria

After determining the identities of the individual isolates to the lowest possible taxonomic level (i.e., species or genus), we randomly selected individual strains to represent each taxon. The selected strains were inoculated into 10 mL lysogeny broth medium, and incubated for 24–36 h, until they reached an OD_600_ of ∼1.0–1.5. Once this concentration was achieved, the liquid medium was removed by centrifugation at 8000 ×*g* and 4°C for 10 min, and the resulting pellets were washed once with 10 ml sterile PBS and re-suspended in 10 mL minimal medium (Voigt et al., 2007). The cultures were then incubated at 28°C for 8 h to allow for the depletion of amino acids, after which the cell concentration of the cultures was determined using a Petroff–Hausser counting chamber and the cultures were standardized to 10^8^ cells/mL. Next, 1 mL of each of the standardized bacterial cultures was inoculated into individual brown bottles (250 mL) that contained 100 mL of nutrient broth with 200 mg/L tryptophan, and the cultures were incubated in a rotary shaker at 28°C with a rotational speed of 150 rpm in the dark. Cell viability was quantified using the plate counting method with appropriate dilutions. After 36 h, the bacterial cultures were centrifuged and the supernatants were diluted 1:1 with methanol, and the resulting mixtures were passed through a 0.22-μm filter and used for preliminary screening of melatonin and TEE production.

To investigate the potential conversion of L-tryptophan to melatonin in the bacterial strain that produced the highest concentration of melatonin, we measured the concentrations of several intermediate molecules of melatonin biosynthesis in its culture medium. The strain was cultivated using the procedure described above. In addition, every 6 h, we sampled the bacterial cultures, centrifuged the samples at 8000 ×*g* for 10 min, and diluted the supernatant 1:1 with methanol. Then, after being passed through a 0.22-μm filter, the resulting mixtures were analyzed for tryptamine, 5-hydroxytryptophan, serotonin, *N*-acetylserotonin, and melatonin contents using UPLC-MS/MS.

### Colonization Assay

To determine whether the melatonin-producing endophytic bacteria could influence the endogenous melatonin level in roots, grapevine plantlets were inoculated with a bacterial isolate that produced the highest amount of melatonin. The bacterial inoculum was prepared by inoculating the strain into 100 mL nutrient broth, incubating the culture for 24–36 h at 28°C, centrifuging the culture at 6000 ×*g* for 10 min, and re-suspending and standardizing the inoculum to 10^7^–10^8^ cells/mL with 0.9% sterilized saline solution. The standardized inoculum was then used to inoculate 6-week-old tissue-cultured *V. labruscana* ‘Summer Black’ plantlets. The roots of the experimental plantlets were immersed in the bacterial inoculum for 1 min, whereas the roots of the control plantlets were treated with 0.9% sterile saline solution, and afterward, all the plantlets were transferred to 500 mL culture bottles that contained 150 g sterile nutrient soil (Pindstrup, Ryomgaard, Denmark) and 40 mL nutrient-rich water that was prepared with the MS (Murashige and Skoog) basic nutrient medium (Cat# M519; Phytotechnology^2^). The plants were randomly distributed in a greenhouse with a 16-h light/8-h dark cycle at 26°C and irrigated with distilled water (5 mL) every 2 days.

After 20 days of endophyte colonization, we randomly selected 12 plantlets from each of the inoculated and control plantlets, in order to compare their growth, which we assessed by measuring root length, root fresh weight (FW), plant height, and chlorophyll content. The chlorophyll content of fully expanded leaves was analyzed using a chlorophyll ELISA Kit, according to the manufacturer’s instructions (Lvyuan, Beijing, China). In addition, the roots were also sampled to determine counts of viable bacteria in the roots of inoculated and control plants, as described in the “Isolation of endophytic bacteria from grapevine roots” section above, and the plate counts of viable bacteria were considered an indicator of bacterial invasion capacity.

Meanwhile, the rest of plantlets were assigned to one of four experimental treatment groups: (i) inoculated plantlets subject to salt stress; (ii) control plantlets subject to salt stress; (iii) inoculated plantlets subject to drought stress; and (iv) control plantlets subject to drought stress. Briefly, 20 mL NaCl solutions (60 or 120 mM) were applied to a series of inoculated and control plantlets in order to simulate salt stress, and 20 mL 10% PEG-6000 solution was added to another series of inoculated and control plantlets in order to simulate drought stress, with 12 plantlets per treatment. After 4 days, the roots were sampled from each plantlet at 9–10:00 AM and ground into powder using liquid nitrogen in individual mortars, and 0.5 g of each root powder was extracted with 2 mL of methanol, as described previously (Boccalandro et al., 2011). The resulting extracts were mixed with 2 mL ultrapure water, centrifuged, passed through a 0.22-μm filter, and stored in amber vials for analysis of 5-hydroxytryptophan, tryptamine, serotonin, *N*-acetylserotonin, and melatonin *via* UPLC-MS/MS.

The H_2_O_2_ levels of the roots were measured according to Patterson et al. (1984), and superoxide production was estimated using the method of Elstner and Heupel (1976). In addition, we assessed the extent of lipid peroxidation in the roots by measuring the MDA content as described by Zhao et al. (2013) with little modification. Briefly, 0.1 g of each root powder was extracted with 1 mL of 10% (w/v) trichloroacetic acid (TCA), vortexed, and centrifuged at 8000 ×*g* for 10 min. Then, 0.2 ml of each supernatant was mixed with 0.2 ml 0.5% (w/v) thiobarbituric acid (TBA) in 20% (w/v) TCA. The mixtures were heated at 100°C for 20 min, cooled, and centrifuged at 8000 ×*g* for 10 min. Absorbances were read at 440, 532, and 600 nm, and the MDA concentration (nmol/g FW) was calculated according to the formula: [6.45 × (A532 – A600) – 0.56 × A450] × V/W, where V (mL) is the volume of the tissue extract, and W (g) is the FW.

### RNA Extraction and Quantitative Real-Time PCR Analysis

Total RNA was isolated from the root tissue of stress-induced plantlets, following the CTAB method (Reid et al., 2006), and the resulting RNA was treated with DNase I and converted to cDNA using the PrimeScript RT reagent Kit with gDNA Eraser (Takara, Dalian, China).

Three TDC homologs, which shared conserved functional domains and >30% homology with the amino acid sequence of rice tryptophan decarboxylase (GenBank No. XP_015648768), were identified by searching the non-redundant protein and nucleotide sequence data of grapevine (*Vitis Vinifera* L.) at the National Center for Biotechnology Information (National Institutes of Health, Bethesda, MD, USA), using the tBLASTn, BLASTp, and PSI-BLAST programs (Altschul et al., 1997); and sequence of the grapevine serotonin *N*-acetyltransferase gene (*VvSNAT*; GenBank No. XM_002266325) was previously predicted by Byeon et al. (2014a). The expression levels of all four genes in the roots of stress-induced plantlets were determined using quantitative real-time PCR (qRT-PCR) analysis with a Roche 480 light cycler System and SYBR Fast qPCR Mix (TaKaRa, Dalian, China). All primers were designed using the NCBI Primer-BLAST service^3^ (**Table 1**), and the qRT-PCR amplification was performed with the following conditions: 95°C for 5 min, followed by 40 cycles of 95°C for 5 s, 60°C for 10 s, and 72°C for 15 s; and melting curve analysis was performed using 95°C for 5 s, 60°C for 1 min, 97°C continuously, and then 40°C for 30 s. All the qRT-PCR reactions were performed in triplicate, and the expression levels of the target genes were normalized using the *EF1-α* gene as an internal reference.

**Table 1:** Primers used for quantitative real-time PCR.

Gene	Primers sequences (5′–3′)
*EF-1*α	F: GAACTGGGTGCTTGATAGGC
	R: AACCAAAATATCCGGAGTAAAAGA
*VvSNAT*	F: GCCCGTGCTACATCAGATCA
	R: TTTGATGCCCTCTGGGTCAG
*VvTDC*1	F: CTGCCAGATTCCGCACCTAA
	R: TCGCCGCAGGAGAAGTAATC
*VvTDC*2	F: CGGAGCTATGGTGTCGTCAA
	R: TCCCCCAACAATGGCATGAG
*VvTDC*3	F: CCAGAGAAGAAGGGGAAAGCA
	R: GGCTCCTGCAGTACGAGTTG

### UPLC-MS/MS Analysis of Metabolites

Tryptophan-ethyl ester, tryptamine, 5-hydroxytryptophan, serotonin, *N*-acetylserotonin, and melatonin were purchased from Sigma-Aldrich (St. Louis, MO, USA). Ultrapure water was produced using a Millipore Milli-Q ultrapure water purification unit (Millipore, Bedford, MA, USA). Other solvents, including methanol and formic acid (HPLC grade), were purchased from Merck (Darmstadt, Germany). Stock solutions were prepared by dissolving 10 mg of each standard in 1 mL methanol under low light conditions; the solutions were then stored at -80°C to prevent degradation. Fresh working solutions were prepared in a methanol:water solution (50:50, v/v).

Quantitative detection was conducted using a UHPLC-ESI-QQQ-MS (Agilent 1290 and 6460 triple quadrupole mass spectrometry series; Agilent Corporation, Santa Clara, CA, USA). In the solvent system, Milli-Q water that contained 0.1% (v/v) formic acid was used as eluent A, and methanol was used as eluent B. The analytes were separated using an Agilent ZORBAX Eclipse XDB-C18, Rapid Resolution HT column (1.8 μm, 3.0 mm × 50 mm) at 42°C with linear elution gradient protocols of 0–6 min, 5–55% B, 6–15 min, 55–100% B, at 0.2 mL/min flow rate. Next, 100% B was kept constant for 2 min and the column was re-equilibrated for 5 min using the initial solvent composition. The injection volume was 1 μL. The metabolites were quantitatively detected using the multiple reactions monitoring mode under unit mass-resolution conditions (tryptamine *m/z* 161→144, 5-hydroxytryptophan *m/z* 221→204, serotonin *m/z* 177→160, *N*-acetylserotonin *m/z* 219→160, and melatonin and TEE *m/z* 233→174) in positive ion mode. To quantify the analytes, we constructed eight-point calibration curves, using diluted working solutions of external standards. All points on the curves represented the average of three independent determinations. The linearity of the calibration graphs was determined using regression analysis. The limits of detection (LOD) were calculated based on the S/N ratio of 3:1. The limits of quantitation (LOQ) were defined as the lowest level that had an S/N ratio of 10:1. All the investigated analytes displayed excellent linearity, with correlation coefficients (*R*^2^) ranging from 0.9975 to 0.9988 (**Table 2**).

**Table 2:** UPLC-MS/MS quantitation data for six analytes.

Analyte	Linearity range (ng/mL)	*R*^2^	LOD (ng/mL)	LOQ (ng/mL)
Tryptamine	0.24–24	0.9975	0.08	0.24
5-Hydroxytryptophan	0.45–45	0.9984	0.15	0.45
Serotonin	0.36–360	0.9988	0.12	0.36
*N*-acetylserotonin	0.18–18	0.9985	0.06	0.18
Melatonin	0.12–1.2	0.9984	0.04	0.12
Tryptophan-ethyl ester	0.1–10	0.9979	0.03	0.1

*LOD, limit of detection; LOQ, limit of quantification.*

### Statistical Analysis

For each experiment, the results were expressed as the mean ± standard deviation of data from 3–12 replicates. Statistical evaluation was performed using one-way ANOVA, followed by Tukey’s test for the data of preliminary screening of melatonin production and Student’s *t*-test for the colonization assay. All the statistical analyses were performed using SPSS (version 19.0; IBM, Armonk, NY, USA), and a *P*-value of <0.05 was considered statistically significant.

## Results

### Melatonin and TEE Production by Endophytic Bacteria from Grapevine Roots

No colonies grew on the plates that were inoculated with water from the final washing step of the root sterilization procedure, suggesting that the surface sterilization procedure was effective. For the remaining plates, the highest bacterial count (5.75 ± 0.26 log10 CFU/g FW) was detected on those inoculated with the homogenized roots of *V. labruscana* ‘Summer Black,’ followed by those inoculated using *V. vinifera* ‘Cabernet Sauvignon’ roots (5.23 ± 0.18 log10 CFU/g FW) and *V. amurensis* ‘Changbai 9’ roots (4.89 ± 0.22 log10 CFU/g FW). A total of 98 endophytic bacteria strains were isolated from the surface-sterilized roots, and 16S rRNA sequences were amplified from each strain, whereas *gyrB* sequences were only amplified from 64. Based on comparison with related sequences deposited in the GenBank DNA database, we identified seven different bacterial genera, which included *Agrobacterium*, *Bacillus*, *Variovorax*, *Pseudomonas*, *Streptomyces*, S*phingomonas*, and *Ensifer*.

**FIGURE 1:**
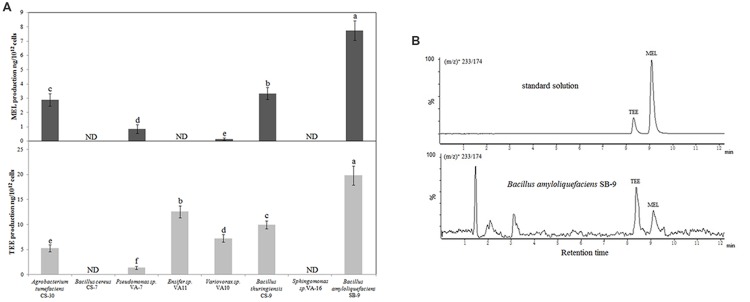
**UPLC-MS/MS detection of melatonin and TEE production by various endophytic bacteria from grapevine roots. (A)** Melatonin and TEE levels in the culture media of the endophytic bacteria after 36 h of incubation in nutrient broth containing 200 mg/L of tryptophan. Values represent means ± SD (*n* = 3), and different letters indicate significant differences at *P* < 0.05, according to Tukey’s test. ND, not detected. **(B)** UPLC-MS/MS chromatograms of standard solutions of two target analytes and a sample from a *Bacillus amyloliquefaciens* SB-9 culture at the transitions of (*m/z*)^+^ 233/174.

The *Streptomyces* strains were excluded from the screening of melatonin- and TEE-producing abilities, owing to their abnormal growth and low biomass in nutrient broth. We randomly screened eight endophytic bacterial strains that represented eight species (**Table 3**). With the exception of *B. cereus* CS-17 and *Sphingomonas sp*. VA-16, all the investigated strains secreted tryptophan derivatives into the medium and exhibited species-specific levels of production (**Figure 1A**). Five of the strains secreted melatonin *in vitro*, and the highest level (7.75 ng/10^12^ cells; 0.87 ng/mL; cell count, 11.15 log10 CFU/mL) was produced by *B. amyloliquefaciens* SB-9, followed by *B. thuringiensis* CS-9 (3.33 ng/10^12^ cells; 0.53 ng/mL; cell count, 11.20 log10 CFU/mL) and *Agrobacterium tumefaciens* CS-30 (2.90 ng/10^10^ cells; 0.22 ng/mL; cell count, 10.88 log10 CFU/mL). TEE has previously been considered as one of the melatonin isomers, and this compound produced the same fragmentation pattern of melatonin using the triple quadrupole mass spectrometry (**Figure 1B**). We found that six strains, including all the melatonin-producing isolates, were able to produce TEE, with amounts ranging from 0.24 to 19.83 ng/10^12^ cells.

**Table 3:** Endophytic bacterial strains screened for melatonin production ability.

Species	Code	Origin	GenBank accession no.	
			16S rRNA gene	*gyrB*
*Agrobacterium tumefaciens*	CS-30	Cabernet Sauvignon	KU522188	KX346711
*Bacillus thuringiensis*	CS-9		KU522196	KX346714
*B. cereus*	CS-17		KU522189	KX346713
*B. amyloliquefaciens*	SB-9	Summer Black	KX346710	KX346712
*Variovorax* sp.	VA10	Changbai 9	KX065462	—
*Pseudomonas* sp.	VA-7		KU522198	KX423685
*Ensifer* sp.	VA11		KX065463	—
*Sphingomonas* sp.	VA-16		KU522199	—

*—, no PCR product.*

### Characterization of Melatonin Formation in *B. amyloliquefaciens* SB-9

**FIGURE 2:**
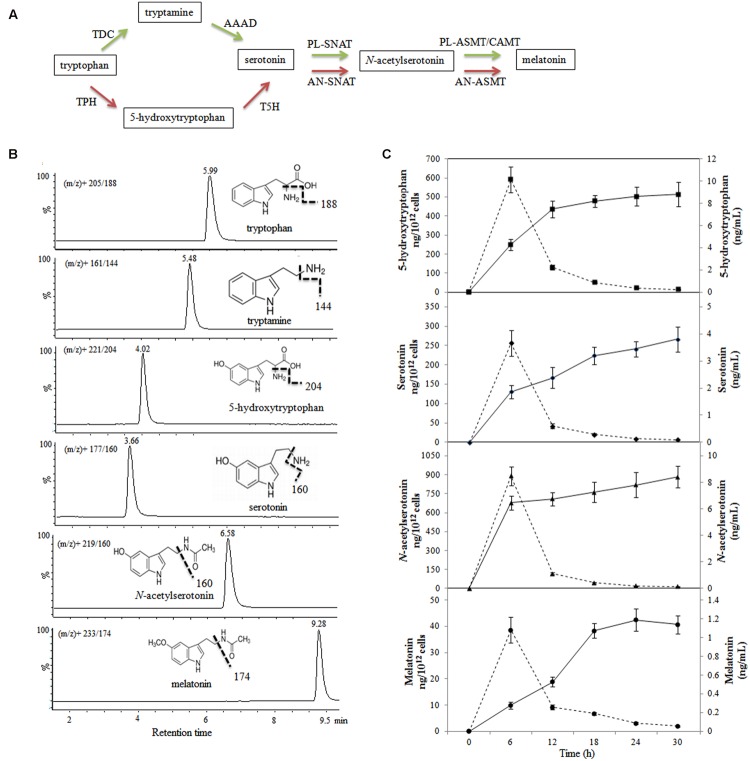
**Evolution of metabolite production in melatonin biosynthesis pathway in *Bacillus amyloliquefaciens* SB-9. (A)** Melatonin biosynthesis pathway in animals and plants. Red arrows indicate the animal pathway, and green arrows indicate the plant pathway. TPH, tryptophan hydroxylase; AAAD, aromatic amino acid decarboxylase; TDC, tryptophan decarboxylase; T5H, tryptamine 5-hydroxylase; AN-SNAT, animal serotonin *N*-acetyltransferase; PL-SNAT, plant serotonin *N*-acetyltransferase; AN-ASMT, animal *N*-acetylserotonin *O*-methyltransferase; PL-ASMT, plant *N*-acetylserotonin *O-*methyltransferase; CAMT, caffeic acid *O-*methyltransferase. **(B)** Chemical structures and UPLC-MS/MS chromatograms of five standards (40–60 ng/mL). **(C)** Levels of metabolites involved in the melatonin biosynthesis in a *B. amyloliquefaciens* SB-9 culture grown in medium containing 200 mg/L tryptophan. The levels are expressed as ng/mL (solid line) and ng/10^12^ cells (dotted line), and the data points represent the means ± SD (*n* = 3).

The melatonin synthetic pathways of animals and plants have been reported previously (**Figure 2A**); however, the mechanism of melatonin synthesis in bacteria is currently unknown. We detected 5-hydroxytryptophan, serotonin, *N*-acetylserotonin, and melatonin after 6 h of incubation; however, we did not find tryptamine during the incubation. The concentrations of 5-hydroxytryptophan, serotonin, and *N*-acetylserotonin increased throughout the incubation period, and reached maximum values of 8.82 ± 1.08, 3.81 ± 0.46, and 8.41 ± 0.82 ng/mL, respectively, after 30 h (**Figure 2**), whereas the concentration of melatonin reached a maximum value of 1.19 ± 0.12 ng/mL after 24 h of incubation and declined slightly thereafter. When the results were expressed as ng/10^12^ viable cells, the production capacity for all the investigated metabolites peaked at 6 h (cell number, 9.87 log10 CFU/mL) and, thereafter, declined with increasing cell density (11.76 log10 CFU/mL at 30 h).

### Growth Responses of *V. labruscana* ‘Summer Black’ Plantlets Treated with *B. amyloliquefaciens* SB-9

**FIGURE 3:**
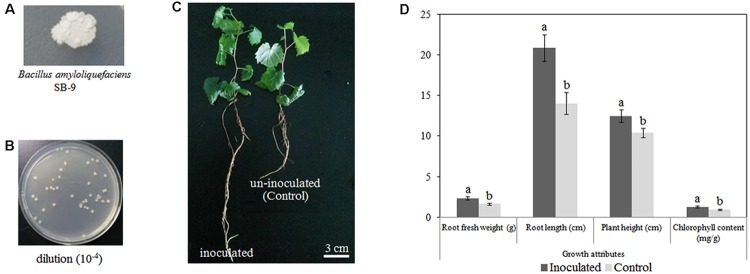
**Colonization of *Vitis labruscana* ‘Summer Black’ plantlets by *Bacillus amyloliquefaciens* SB-9. (A)** Colony morphotype of *B. amyloliquefaciens* SB-9. **(B)** Colony count of bacteria that were re-isolated from inoculated plantlets. **(C)** Morphological differences between inoculated and control plantlets. **(D)** Effects of *B. amyloliquefaciens* SB-9 inoculation on the growth attributes of grapevine plantlets. The values represent means ± SD (*n* = 12). Different letters indicate significant differences (*P* < 0.05) between inoculated and un-inoculated (control) plantlets, according to Student’s *t*-test.

Based on its high melatonin-producing capacity, we selected *B. amyloliquefaciens* SB-9 for the inoculation of grapevine plantlets. We observed no symptoms of pathogenicity in the inoculated *V. labruscana* ‘Summer Black’ plantlets. At 20 days after inoculation, *B. amyloliquefaciens* SB-9 was successfully re-isolated from inoculated roots, and its identity was verified *via* sequencing of the 16S rRNA region. In addition, the colonies recovered from the inoculated seedling roots exhibited a single morphotype (**Figure 3A**), and the population density was 5.74 ± 0.22 log10 CFU/g FW (**Figure 3B**), which indicated a high level of colonization, whereas no colonies were isolated from the control plant roots. Furthermore, we also observed that inoculation with *B. amyloliquefaciens* SB-9 significantly promoted the growth of the grapevine plantlets. In fact, the root length, plant height, FW, and leaf chlorophyll content of the inoculated plantlets were 48.58, 19.46, 41.82, and 41.76% greater, respectively, than those of the control plantlets (**Figures 3C,D**), which indicates that the strain can be regarded as a valuable plant growth-promoting rhizobacteria.

### Effect of *B. amyloliquefaciens* SB-9 on Levels of Endogenous Melatonin and Its Intermediates in Roots under Stress

**FIGURE 4:**
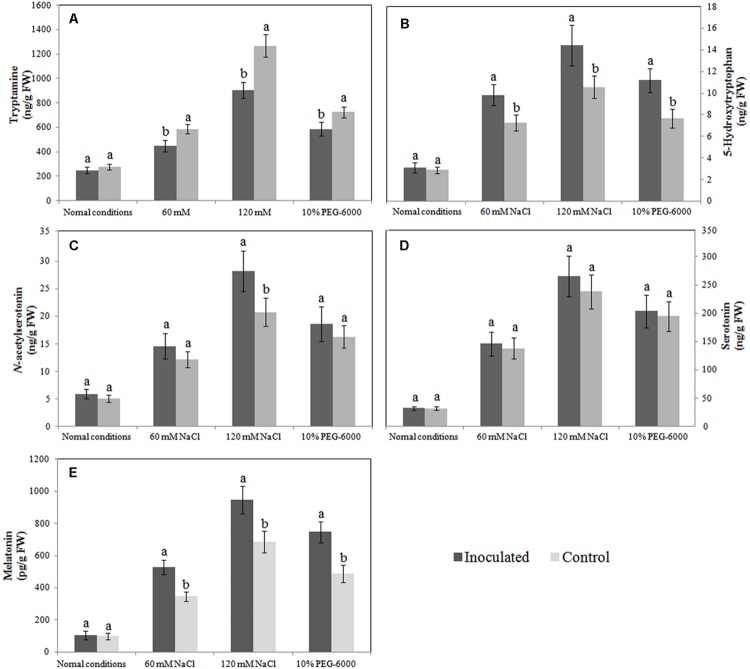
**Effects of *Bacillus amyloliquefaciens* SB-9 inoculation on levels of endogenous tryptamine (A), 5-hydroxytryptophan (B), *N*-acetylserotonin (C), serotonin (D) and melatonin (E) in the roots of salt- and drought-stressed *Vitis labruscana* ‘Summer Black’ plantlets.** Values represent means ± SD (*n* = 12). Different letters indicate significant differences (*P* < 0.05) between inoculated and un-inoculated (control) plantlets, according to Student’s *t*-test.

When the roots of inoculated and control plantlets were subjected to salt or drought stress, they responded by synthesizing melatonin and its intermediates (tryptamine, 5-hydroxytryptophan, serotonin, and *N*-acetylserotonin), albeit to different extents. We found that melatonin synthesis was similar in the inoculated and control plants under normal growth conditions; however, after exposure to salt or drought stress, the melatonin levels in the roots of inoculated plantlets were 52.61, 37.90, and 53.07% higher, respectively, than those in the roots of control plantlets (**Figure 4E**). Similarly, in the absence of abiotic stresses, 5-hydroxytryptophan levels in inoculated plants and control plants were similar, but after NaCl or 10% PEG 6000 treatment, the synthesis of 5-hydroxytryptophan in the roots of inoculated plantlets was significantly higher than that in the roots of control plants (**Figure 4B**). A similar trend was also observed for *N*-acetylserotonin, and in salt-stress plantlets (120 mM only), its level was significantly upregulated in inoculated plantlets, when compared to control plants (**Figure 4C**); however, the tryptamine levels in the roots of inoculated plantlets were significantly lower than those in the roots of control plants after exposure to abiotic stresses (**Figure 4A**), and the serotonin levels in inoculated and control plantlets were statistically similar (*P* > 0.05; **Figure 4D**). Therefore, it is likely that the synthesis of melatonin and its intermediates in the roots of plants exposed to abiotic stress is influenced by *B. amyloliquefaciens* SB-9 colonization.

### Effect of *B. amyloliquefaciens* SB-9 on Stress-Induced Oxidative Damage

**FIGURE 5:**
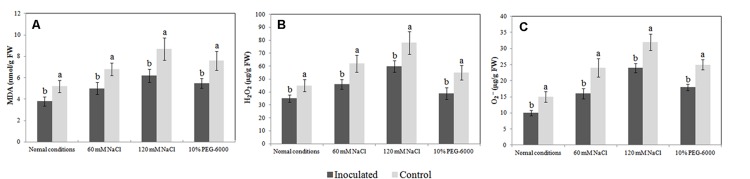
**Effects of *Bacillus amyloliquefaciens* SB-9 inoculation on the levels of malondialdehyde (A), H_2_O_2_ (B), and O_2_^-^ (C) in the roots of salt- and drought-stressed *Vitis labruscana* ‘Summer Black’ plantlets.** MDA, malondialdehyde. Values represent means ± SD (*n* = 12). Different letters indicate significant differences (*P* < 0.05) between inoculated and un-inoculated (control) plantlets, according to Student’s *t*-test.

The MDA level in plant tissue is an indicator of lipid peroxidation status, and it is accompanied by ROS production (H_2_O_2_ and O_2_^-^). In the absence of abiotic stresses, the MDA level was markedly lower in the roots of inoculated plantlets than in the roots of un-inoculated plantlets (3.8 nmol/g FW vs. 5.2 nmol/g FW). Additionally, inoculated and un-inoculated plantlets exposed to salt and drought stress had increased MDA levels; however, the MDA levels were significantly lower in the roots of endophyte-associated plantlets than in the roots of un-inoculated plantlets (**Figure 5A**). We observed similar trends in ROS production between un-inoculated and inoculated plantlets (**Figures 5B,C**). Our findings indicate that colonization with *Bacillus amyloliquefaciens* SB-9 counteracted the adverse effects of abiotic stress by reducing the production of MDA and ROS.

### Relative Expression of Melatonin Synthesis Genes

**FIGURE 6:**
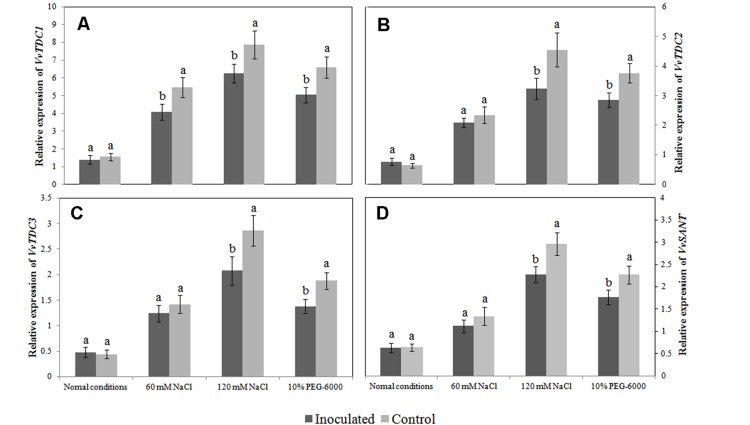
**Effects of *Bacillus amyloliquefaciens* SB-9 inoculation on the relative expression of the *VvTDC1* (A), *VvTDC2* (B), *VvTDC3* (C), and *VvSNAT* (D) genes in the roots of salt- and drought-stressed *Vitis labruscana* ‘Summer Black’ plantlets.** The expression levels were normalized according to the expression of grapevine *EF1-*α mRNA. Values represent means ± SD (*n* = 12). Different letters indicate significant differences (*P* < 0.05) between inoculated and un-inoculated (control) plantlets, according to Student’s *t*-test.

The *VvTDC1*, *VvTDC2*, and *VvTDC3* genes are located on grapevine chromosomes 7, 10, and 4, respectively, and their predicted amino acid sequences possessed 48.50, 47.87, and 47.49 % homology with rice tryptophan decarboxylase (*TDC1*). Similar to the pattern observed for endogenous melatonin levels, we observed that the relative expression levels of *VvTDC1*, *VvTDC2*, *VvTDC3*, and *VvSNAT* in the roots of control plantlets were significantly upregulated by both salt and drought stress (**Figure 6**), again suggesting that melatonin synthesis is stress-inducible. Interestingly, the transcript levels of the *VvTDCs* and *VvSNAT* in the roots of SB-9-inoculated plants were also increased by both salt and drought stress; however, the extent of upregulation for these genes was significantly lower (*P* < 0.05) than that of control plants when they were subject to identical stressors, with the exception of *VvTDC1* under 60 mM salt stress.

## Discussion

Melatonin was previously identified in the primitive photosynthetic bacterium *Erythrobacter longus* (Tilden et al., 1997) and in the gram-negative bacterium *Escherichia coli* (Hardeland and Poeggeler, 2003); however, few other bacteria are known to produce melatonin. In the present study, we demonstrated that endophytic bacteria, such as *A. tumefaciens* and *B. amyloliquefaciens*, are capable of secreting melatonin into extracellular media (**Figure 1A**). In addition, we also found that the level of melatonin in seedling roots was greater when the roots were colonized by *B. amyloliquefaciens* SB-9 and subjected to salt or drought stress (**Figure 2A**). These findings are in accordance with those of Arnao and Hernández-Ruiz (2015), who proposed an association between beneficial endophytes and the enhanced melatonin levels in their host plants. However, we cannot be sure that the enhanced levels of endogenous melatonin were derived from the endophytic bacteria. Alternatively, root cells might utilize intermediate metabolites of melatonin that are produced by endophytic bacteria. In fact, in the present study, *B. amyloliquefaciens* SB-9 secreted serotonin and *N*-acetylserotonin.

The melatonin biosynthesis pathway of plants was recently described (Byeon et al., 2014b) and was shown to differ markedly from that of vertebrates (Tan et al., 2014). One difference is that plants initially decarboxylate tryptophan to form tryptamine and subsequently hydroxylate tryptamine to form serotonin, whereas vertebrates initially hydroxylate tryptophan to form 5-hydroxytryptophan. In the present study, we failed to detect tryptamine in the *B. amyloliquefaciens* SB-9 culture; however, the concentration of 5-hydroxytryptophan increased throughout the incubation period (**Figure 2C**). This suggests that the first step of melatonin biosynthesis in the endophytic bacterium *B. amyloliquefaciens* SB-9 may be similar to that in vertebrates. However, the entire melatonin biosynthesis pathway remains to be elucidated, and further studies of the genes involved in the melatonin biosynthesis pathway of *B. amyloliquefaciens* SB-9 are required.

Endophytic bacteria play an important role in promoting plant growth; however, their influence might be the result of cumulative effects from the various bioactivities of individual endophytes. Indeed, N_2_-fixing bacteria are also capable of producing IAA (Pedraza et al., 2004), solubilizing phosphate, and releasing amino acids (Liba et al., 2006). Furthermore, it has also been reported that IAA and gibberellins frequently occur simultaneously in culture broth (Piccoli et al., 2011), which indicates that individual endophytic bacterium may be capable of synthesizing multiple phytohormones. All of the above-mentioned factors are beneficial to plant growth. We found that endophytic bacteria produced an additional growth regulator, melatonin, which was previously reported to stimulate lateral root and shoot growth in several plants, even at low concentrations (Chen et al., 2009; Park and Back, 2012; Bajwa et al., 2014; Wei et al., 2014; Hernández-Ruiz and Arnao, 2016). In the present study, plant height, FW, leaf chlorophyll content, root length, and number of lateral roots (data not shown) were all enhanced by SB-9 colonization. Although we are not sure whether the other *B. amyloliquefaciens* strains could enhance the endogenous melatonin level in roots, this growth-promoting activity has also been confirmed by other researchers (Idriss et al., 2002; Zhang et al., 2015), Until now, however, we have no direct evidence that the SB-9-derived melatonin enhancement played a role in the growth attributes we measured. This is especially true since the growth promotion, including main/lateral roots development, were likely derived from the combined effects of plant growth-promoting rhizobacteria (PGPR), such as nitrogen fixation, phosphorus solubilization, the production of 1-aminocyclopropane-1-carboxylate (ACC) deaminase or other PGPR-induced physical and chemical (gibberellin, auxin, cytokinin, and unknown metabolites) changes in plants. Further studies, using a mutant that is unable to increase the endogenous melatonin level of roots, are needed to determine the correlation between the enhanced level of melatonin in endophytic bacteria and the promotion of growth in their host plants.

Recent studies have suggested that some plants accumulate melatonin as a defense against a variety of environmental abiotic stressors, such as salt (Mukherjee et al., 2014), chemical agents (Arnao and Hernández-Ruiz, 2009b; Byeon et al., 2015), low temperature (Shi et al., 2015), and drought (Arnao and Hernández-Ruiz, 2013a,b). The results of the present study concur with those of previous reports, and we also found that stress-induced melatonin synthesis was accompanied by the upregulation of several *VvTDCs* and *VvSNAT*, as well as the increased production of melatonin intermediates, such as tryptamine, 5-hydroxytryptophan, serotonin, and *N*-acetylserotonin (**Figure 4**).

Endophytes have mostly been reported to counteract the adverse effects of stress by reducing the production of MDA and ROS in plants (Jungwook et al., 2009; Khan et al., 2012). This behavior is likely derived from the combined effects of endophytes, such as the enhancement of plant antioxidant enzyme (POD, CAT, POD and APX) activities and the production of phenolic compounds or other antioxidant compounds (Torres et al., 2012). We found that, in grapevine plantlets exposed to salt or drought stress, the production of MDA and ROS, as well as the transcript levels of the grapevine *VvTDC*s and *VvSNAT* in inoculated roots were relatively lower than those in un-inoculated controls. Therefore, it seems logical that the endogenous levels of melatonin and its intermediates in inoculated roots could be also lower than those in the un-inoculated controls. However, only tryptamine levels exhibited this trend, whereas levels of the other intermediates in the roots of inoculated plants were similar or higher than those in the un-inoculated controls. We speculate that the lower expression of *VvTDCs* and *VvSNAT* could be associated with lower oxidative damage in the roots of inoculated plantlets (**Figures 5** and **Figures 6**) because the transcript levels of melatonin synthesis genes were reported to show a positive correlation with ROS levels caused by abiotic stress (Li et al., 2014); however, the higher endogenous 5-hydroxytryptophan, *N*-acetylserotonin and melatonin levels, as well as the statistically similar serotonin level, in the roots of these plantlets was due to SB-9 colonization, which may promote the production of these compounds via supplementary bacterial melatonin biosynthesis, a possible exchange of metabolites between plants and the strain, or additional promoting factors produced by the strain. In fact, in the present study, *B. amyloliquefaciens* SB-9 was able to secrete these compounds *in vivo*, except tryptamine. In primitive bacteria, melatonin is thought to function as an antioxidant and free radical scavenger that reduces the harmful effects of ROS (Tan et al., 2014). This is the primary role that was reported in both animals and plants (Tan et al., 2002, 2003; Reiter et al., 2005). Therefore, based on these observations, if abiotic stress induces a burst of ROS in the internal tissues of host plants, the living conditions for root-inhabiting microbes presumably become toxic, and as a result, endophytes upregulate their melatonin biosynthesis accordingly, in order to alleviate ROS-induced cell damage.

In summary, the findings of the present study demonstrate that the melatonin-producing ability of endophytic bacteria and the potential application of these bacteria in promoting endogenous melatonin level in plants. However, it remains unclear (i) whether the enhanced levels of endogenous melatonin in roots were derived from production by endophytic bacteria; (ii) whether endophytic fungi produce melatonin; (iii) whether fruit-colonizing endophytes enhance the melatonin levels of fruit tissue, especially in grapevines; (iv) whether any endophytic bacteria, even those with low melatonin-producing abilities, are able to enhance the endogenous melatonin levels in their host plants by providing intermediate metabolites; or (v) which specific internal and external elements influence melatonin production in endophytes and their host plants? Therefore, more comprehensive and detailed investigations are needed to characterize the role of melatonin biosynthesis in naturally occurring symbiotic relationships.

## Author Contributions

YL, CY, JJ, YS, and YQ conceived the study; JJ and YM performed the experiments, analyzed the data, and wrote the manuscript; SC analyzed the UPLC-MS/MS data; CL provided suggestions and revised the manuscript. All authors approved the final manuscript and agreed to be accountable for all aspects of the work, i.e., ensuring that questions related to the accuracy or integrity of any part of the work are appropriately investigated and resolved.

## Conflict of Interest Statement

The authors declare that the research was conducted in the absence of any commercial or financial relationships that could be construed as a potential conflict of interest.

## Footnotes

1 http://www.ncbi.nlm.nih.gov/BLAST

2 http://www.phytotechlab.com/

3 http://www.ncbi.nlm.nih.gov/tools/primer-blast/

## Abbreviations

IAA: indole-3-acetic acid
MDA: malondialdehyde
ROS: reactive oxygen species
TEE: tryptophan-ethylester.
